# 3D Differentiation of Neural Stem Cells in Macroporous Photopolymerizable Hydrogel Scaffolds

**DOI:** 10.1371/journal.pone.0048824

**Published:** 2012-11-07

**Authors:** Hang Li, Asanka Wijekoon, Nic D. Leipzig

**Affiliations:** Department of Chemical and Biomolecular Engineering, The University of Akron, Akron, Ohio, United States of America; University of Milan-Bicocca, Italy

## Abstract

Neural stem/progenitor cells (NSPCs) are the stem cell of the adult central nervous system (CNS). These cells are able to differentiate into the major cell types found in the CNS (neurons, oligodendrocytes, astrocytes), thus NSPCs are the mechanism by which the adult CNS could potentially regenerate after injury or disorder. Microenviromental factors are critical for guiding NSPC differentiation and are thus important for neural tissue engineering. In this study, D-mannitol crystals were mixed with photocrosslinkable methacrylamide chitosan (MAC) as a porogen to enhance pore size during hydrogel formation. D-mannitol was admixed to MAC at 5, 10 and 20 wt% D-mannitol per total initial hydrogel weight. D-mannitol crystals were observed to dissolve and leave the scaffold within 1 hr. Quantification of resulting average pore sizes showed that D-mannitol addition resulted in larger average pore size (5 wt%, 4060±160 µm^2^, 10 wt%, 6330±1160 µm^2^, 20 wt%, 7600±1550 µm^2^) compared with controls (0 wt%, 3150±220 µm^2^). Oxygen diffusion studies demonstrated that larger average pore area resulted in enhanced oxygen diffusion through scaffolds. Finally, the differentiation responses of NSPCs to phenotypic differentiation conditions were studied for neurons, astrocytes and oligodendrocytes in hydrogels of varied porosity over 14 d. Quantification of total cell numbers at day 7 and 14, showed that cell numbers decreased with increased porosity and over the length of the culture. At day 14 immunohistochemistry quantification for primary cell types demonstrated significant differentiation to the desired cells types, and that total percentages of each cell type was greatest when scaffolds were more porous. These results suggest that larger pore sizes in MAC hydrogels effectively promote NSPC 3D differentiation.

## Introduction

Tissue engineering offers new medical therapies to regenerate diseased or damaged tissues and aims to create three-dimensional (3D) scaffolds to accommodate cells and guide their growth both *in vitro* and *in vivo* [1], [2], [3]. Tissue engineered scaffolds should have appropriate chemical and physical properties to promote adhesion, proliferation, and differentiation that are specific for each cell and tissue type. These scaffolds must be biocompatible for implantation and including interconnecting pores of appropriate scale has been shown to favor tissue integration and vascularization [4], [5]. In scaffold-based tissue engineering, a scaffold must be permeable to supply adequate oxygen and nutrients to cells throughout the construct, as well as to remove waste products [6]. Within a scaffold, mass transport of oxygen, nutrients and waste is mainly achieved by diffusion, so it is critical to create three dimensional porous scaffolds to facilitate this process [7], [8], [9]. Often in larger tissue engineered constructs, diffusion limitations lead to low cellularity in central regions of the scaffolds [8]. Pore structure also significantly affects cell attachment and migration *in vitro*; and scaffold mean pore size is known to influence cell morphology and phenotypic expression [10], [11], [12], [13], [14]. Interconnected macropores of at least 100 µm are important for *in vivo* integration, especially for vascularization [15], [16], [17]. For cell penetration *in vitro*, the optimal interconnection size is over 40 µm. Importantly, nutrient permeability in scaffolds increases [16] with increasing pore size [18]. Porous three-dimensional scaffolds are typically fabricated from synthetic and naturally biodegradable polymers [19]. Techniques to create porous three-dimensional scaffolds include gas foaming [20], particulate leaching [21], phase separation [22] and electrospinning [23]. The most common method for creating macroporous scaffolds are based on the addition of templates called porogens or pore forming agents to stiff polymeric scaffolds (e.g. polycaprolactone, polylactic acid, polyglycolic acid, etc.). Generally the porogen is either an organic material that can be combusted away by heating [24], [25] or a soluble additive that leaves pores by dissolution [26], [27]. The dissolution technique can be performed *in vivo* or *in vitro* in the presence of cells if the porogen is biocompatible and does not appreciably alter the cell microenvironment (e.g., local pH, osmolarity, inflammatory particles) and is eliminated from the scaffold in a timely fashion [28], [29], [30], [31]. D-mannitol macroparticles are especially interesting for their biocompatibility, quick dissolution and are non-toxic [29], [31], [32], and have been used in bone cement and bone scaffolds *in vivo* [28], [30], [31].

We are specifically interested in creating tissue engineered scaffolds uniquely tailored for the central nervous system (CNS) and porosity is equally important for CNS constructs. Currently available clinical treatments for diseased or damaged CNS tissue are limited to prevention of further damage and minor pharmacological relief [33]. A serious need exists for treatments that can restore function in the CNS. For example, spinal cord injury (SCI) and the intrinsic inability of this tissue to regenerate highlight the need for regenerative therapies for CNS tissues. A key component of any tissue engineering strategy is cell source; fortunately, populations of stem cells have been identified in the adult CNS that possess the ability to self-renew, generate identical progeny and differentiate into the primary cell types found in the CNS: neurons, oligodendrocytes and astrocytes [34], [35], [36]. These neural stem/progenitor cells (NSPCs) have been isolated from two neurogenic regions of the adult brain, the hippocampus and subventricular zone (SVZ), as well as the spinal cord. Adult SVZ-derived NSPCs have also been isolated from living human patients and demonstrated self-renewal and multipotentiality [37]. With our current technologies this is a difficult and highly invasive procedure, thus, autologous NSPCs are currently an unlikely tissue engineering cell source, however, homologous NSPCs isolated from organ donors are a viable alternative. Additionally, the study of NSPC behavior is important for basic insight while providing knowledge and methods to specify neuronal and glial commitment of adult, embryonic and reprogramed stem cells. NSPCs offer an attractive means of regenerating lost or damaged CNS tissue after disease or injury and we and others have shown that hydrogels are most appropriate as inductive scaffolds since NSPCs, neurons and mature glia prefer very soft substrates (elastic modulus (E) <1 kPa) [38], [39].

The main objectives of this study were to first determine if D-mannitol could be used to enhance the porosity of soft chitosan based photopolymerizable hydrogels, and secondly to determine if NSPCs differentiation is affected by scaffold porosity. To encourage optimal cell-scaffold interactions, a scaffold with a proper microstructure is required. Pore size and porosity are considered as critical factors for cell growth in tissue engineering. Previous studies show that pore size affects many cell activities including adhesion, proliferation, and migration [10], [40], [41]. In this study, D-mannitol crystals were used as a cell compatible porogen to create photopolymerizable 3D hydrogel scaffolds with improved porosity for neural tissue engineering scaffolds. This approach is different from how porosity of hydrogels is typically modulated [20], [21], [22], [23]. After characterizing D-mannitol’s effects on photopolymerizable chitosan hydrogel porosity and its effects on oxygen diffusion, the system was used to encapsulate and guide the differentiation of NSPCs into neurons, oligodendrocytes and astrocytes using differentiation mediums specific to each phenotype. We hypothesize that D-mannitol can be used to create highly porous chitosan based hydrogel scaffolds that will promote cell differentiation under phenotype specific media conditions due to enhanced nutrient diffusion.

## Materials and Methods

### Ethics Statement

The protocols for animal use for this experimentation are currently approved by the Institutional Animal Care and Use Committee (IACUC) at the University of Akron as of January 14, 2010 (IACUC approved protocol number 10-1B). Wistar rats (species *Rattus norvegicus*) that were 6–8 weeks of age were purchased from Charles River Labs (Wilmington, MA) and sacrificed one week after arrival using carbon dioxide, which constitutes a rapid and approved method of euthanasia. Veterinary care was not required for these studies.

### Porous Methacrylamide Chitosan (MAC) Scaffold Preparation

**Figure 1:**
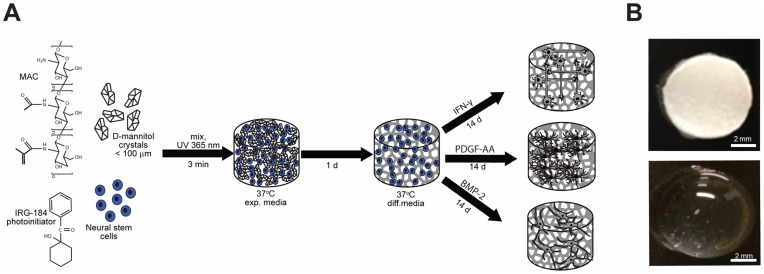
(A) Methodology for creating 3D porous MAC scaffolds and procedure for NSPC culture and differentiation in 3D environments. (B) Images of a 20% D-mannitol scaffold captured immediately after crosslinking and after PBS dissolution for 1 hr at 37°C.

Methacrylamide chitosan (MAC) was synthesized as described previously [42]. Briefly, chitosan (Protosan UP B 80/20, NovaMatrix, Drammen, Norway) was solubilized (3 wt% w/v in acetic acid) overnight then mixed with methacrylic anhydride for 4 hr to synthesize MAC. The resulting solution was dialyzed (MWCO: 12–14,000) against deionized (DI) water for 3 d at room temperature (RT), DI water was changed three times per day. Finally, the solution was freeze dried and stored in the freezer (−20°C) until use. For hydrogel formation, MAC was first dissolved in DI water at 3 wt% and then sterilized by autoclave. We and others have utilized autoclaved chitosan and MAC in previous studies and without noticeable change in properties [42], [43], [44]. Laminin protein (Life Technologies, Grand Island, NY, USA) was covalently bound to MAC at 50 µg/mL by reacting with 5 mM 1-ethyl-3-(3-dimethylaminopropyl) carbo-diimide (EDC; Chem-Impex International, Wood Dale, IL, USA) and 5 mM N-hydroxysulfosuccinimide (sulfo-NHS, Chem-Impex) for 1 hr which covalently linked carboxylic acid containing amino acid residues in laminin to the primary amines of MAC polymers. Photoinitiator solution, 1-hydroxycyclohexyl phenyl ketone (Sigma-Aldrich, St. Louis, MO, USA) 300 mg/mL, in 1-vinyl-2-pyrrolidinone (Sigma-Aldrich,) was added in MAC solution at 3 µL/g (initiator/MAC solution). The photoinitiator was sterilized by 0.2 µm filtration before use D-mannitol (Sigma-Aldrich) was used to modify porosity, this required recrystallization by dissolving in 50% ethanol, crystallizing and sieving to 80–120 µm. MAC macroporous scaffolds (Fig. 1) were created by mixing the MAC scaffolding solution with immobilized laminin with a desired amount of cell suspension (see below), 10X PBS (autoclaved), photoinitiator and varied amounts of D-mannitol crystals (0, 5, 10, 20 wt% mass D-mannitol/total mass of solution) to achieve 2 wt % MAC finally buffered in 1X PBS. This solution was thoroughly mixed and degassed (1 min, 1500 RPM; SpeedMixer DAC 150 FVZ, Hauschild Engineering, Hamm, Germany). 100 µL of the resulting mixture was transferred to a 96-well plate, which provides a cylindrical mold for scaffold formation. Polymerization was achieved by exposure to UV (365 nm) light for 3 min. Appropriate media was added (see section 2.4) and D-mannitol dissolved within 1 hr (Fig. 1B). The scaffolds with D-mannitol changed from opaque to translucent as D-mannitol dissolved. Pore sizes were quantified by cryosectioning unfixed scaffolds at 20 µm on to glass slides (Microm HM 560, Richard Allen Scientific, Kalamazoo, MI, USA), imaging with a microscope (Olympus IX81, Tokyo, Japan) and analyzing (MetaMorph, Sunnyvale, CA, USA). For each slide, 7 random positions from 1 slide were selected to measure pore size for each group (0, 5, 10, 20 wt% D-mannitol) and final values were averaged.

### Oxygen Diffusion Through Scaffolds

**Figure 2:**
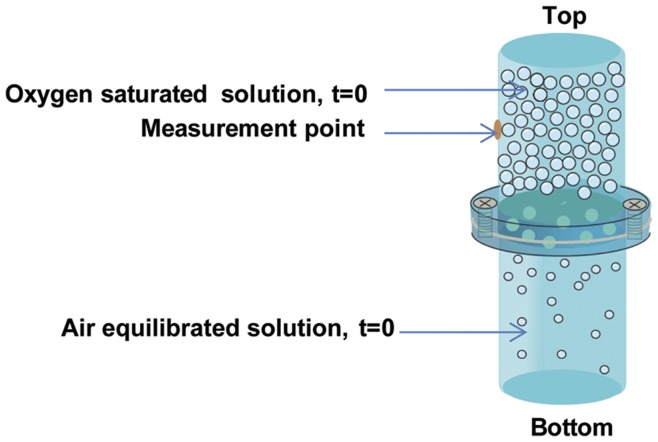
Oxygen diffusion in macroporous MAC scaffolds. Oxygen diffusion device allowing us to measure depletion of oxygen in the top chamber as diffusion occurs through the gel into the oxygen free bottom chamber.

A sealed oxygen diffusion device was constructed out of glass to measure oxygen diffusion through MAC scaffolds (Fig. 2). The top chamber of the device was filled with DI water partially saturated with pure oxygen (purged for 45 min), and the bottom chamber was filled with air-equilibrated DI water. MAC scaffolds with or without D-mannitol were formed in 6 well plates as described above, and then placed in between these two regions of the diffusion device. A Teflon mold was used to prevent radial diffusion, so that water only contacted the top and bottom surfaces of the scaffold, thus oxygen diffusion was limited to the axial direction (Fig. 2). Oxygen concentration was measured by a dissolved oxygen (DO) sensor (PreSens, Regensburg, Germany) every 30 min for 8 hrs in the top chamber to measure oxygen concentration depletion as O_2_ diffused through the scaffold into the bottom chamber. Three scaffolds were tested for each treatment group.

### MAC Hydrogel Stiffness and Swelling Experiments

The mechanical properties of each MAC hydrogel group (0, 5, 10, 20 wt% D-mannitol) were determined with a rheometer (Rheometric Scientific RFS-III, Piscataway, NJ, USA), which computed the complex modulus (G*). The elastic modulus (E) was determined from G* by assuming a poison’s ratio (ν) of 0.5 with the expression E = 2G*(1+ ν) to allow comparison to other published work. In order to estimate the swelling ratio, 100 µL MAC hydrogels (0, 5, 10, 20 wt% D-mannitol) were made and lyophilized. Next their dry mass (M_D_) was measured, followed by swelling of the dry hydrogel scaffolds in 1X PBS at 37°C. Every two hours hydrogels were carefully centrifuged on 50 µm cell strainers (1500 RPM) to remove any PBS clinging to the edges of the scaffolds. The mass after swelling (M_s_) was determined when mass no longer changed and the swelling ratio (Q_M_) was calculated by following equation [45], [46]:
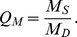



### NSPC Harvest and Cell Culture

NSPCs were harvested from the lateral ventricles of the forebrain of adult rats (Female Wistar, 6–7 weeks old), and the tissue was cut into small pieces and dissociated using a Papain Dissociation System Kit (Worthington Biochemical Corporation, Lakewood, NJ, USA) [38]. NSPCs were expanded as neurospheres in growth medium consisting of neurobasal media (NBM), 2 mM L-glutamine, 100 µg/mL penicillin-streptomycin, B27 (all Life Technologies), 20 ng/mL epidermal growth factor (EGF-recombinant human, Life Technologies), 20 ng/mL basic fibroblast growth factor (bFGF-recombinant human, Life Technologies) and 2 µg/mL heparin (Sigma-Aldrich) in a 37°C, 5% CO_2_ incubator and passaged/expanded weekly. To differentiate NSPCs, the cells can be dissociated, seeded into a suitable environment and cultured in differentiation medium (NBM, L-glutamine, penicillin-streptomycin and B27) containing a growth factor known to favor specific differentiation into either neurons, oligodendrocytes or astrocytes. For our 3D differentiation studies three growth factor treatments were used: 150 ng/mL IFN-γ (for neurons), 25 ng/mL PDGF-AA (for oligodendrocytes) and 20 ng/mL BMP-2 (for astrocytes). The concentrations of INF-γ and PDGF-AA represent saturation concentrations of the growth factors as have been confirmed in previous studies by us and others studying NSPC differentiation to both neurons and oligodendrocytes [47], [48], [49], [50]. In initial experimentation we discovered that 20 ng/mL of BMP-2 was appropriate after simple differentiation experiments on laminin coated glass coverslips. NSPCs were dissociated and cultured in differentiation medium containing increasing concentrations of BMP-2 (0, 5 ng/mL, 10 ng/mL, 20 ng/mL, 40 ng/mL and 80 ng/mL) for 7 d. Samples were fixed then stained for glial fibrillary acidic protein (GFAP), imaged and quantified for the percentage of cells staining positive for GFAP (details provided below in section 2.6). 100% of NSPCs stained positive for GFAP at a BMP-2 concentration of 10 ng/mL to 80 ng/mL (Supplementary Table S1), thus we were confident that 20 ng/mL would be in the saturation range for our 3D experiments. For 3D differentiation experimentation, passage 5 or 6 neurospheres were dissociated and 2×10^6^ cells/g scaffold mixture were added to MAC/D-mannitol scaffolds, then crosslinked into hydrogels as described in section 2.1. Scaffolds were first cultured in growth medium for 1 d (one full media change was performed 1 hr after scaffolds were formed) and then cultured in specific differentiation medium for 7 d (Fig. 1), a half medium change was performed at day 3. At day 7, three scaffolds for each group were fixed with methanol (−20°C) for 10 min in the freezer (−20°C), then washed with PBS. Three scaffolds were also selected for total DNA analysis. At day 14, three scaffolds per group were methanol fixed for immunohistochemistry (IHC) and the remaining scaffolds were used for total DNA analysis. Control groups were included that were first cultured in growth medium (with EGF/FGF) for 1 d and three scaffolds were selected (15 scaffolds total) for total DNA analysis. After day 1, half of the remaining scaffolds (6 scaffolds) were transferred to differentiation media without growth factors (Diff/No GF) and the other scaffolds were maintained in growth media for 14 days. At day 7 and day 14 total DNA analysis was performed for all groups (three scaffolds per group) and at day 14 IHC and quantification was performed for scaffolds both in growth media and Diff/No GF.

### Immunohistochemistry

For IHC, fixed scaffolds were cryosectioned at 20 µm and mounted on slides. The following primary antibodies and dilutions were used for IHC staining: monoclonal mouse anti-β-III tubulin (1∶1000, Covance, Princeton, NJ, USA) identified neurons; monoclonal anti-RIP (Developmental Studies Hybridoma Bank, Iowa City, IA, USA) identified oligodendrocytes; monoclonal mouse anti-GFAP (Cell Signaling, Boston, MA, USA) identified astrocytes; and monoclonal mouse anti-nestin (BD Biosciences, San Jose, CA, USA) were used for staining NSPCs. For staining first 0.1% Triton X-100 (Sigma-Aldrich) in PBS was used for 10 min to permeabilize cell membranes. Then samples were washed 3 times with PBS and blocked with 10% FBS in PBS at RT for 45 min. Samples were washed two times with PBS and incubated with the appropriate primary antibody overnight. Next samples were washed 3 times, each wash ≥ 10 min, and incubated with secondary goat anti-mouse IgG Alexa-Fluor 546 (1∶400, Life Technologies) for 1 hr, then washed with PBS 3 more times (each wash ≥ 15 min). Finally, cell nuclei were stained with10 µM Hoechst 33342 for 7 min, then samples were washed and mounted with Prolong Gold anti-fade reagent (both Life Technologies). Controls where the primary antibody was omitted were performed throughout to determine if any non-specific staining existed. Samples were imaged using a fluorescent microscope (Olympus IX81). For each scaffold (three per group), images of six different non-sequential sections were obtained at 20X magnification. The total cell number was determined by counting intact individual Hoechst 33342 stained cell nuclei in comparison to the number of cells staining positive for the markers of interest. Whole scaffolds were also stained with a modified protocol (double incubation and washing times) and imaged using a multiphoton excitation microscope (Olympus FV1000MPE).

### Quantification of Total Cell Number

To digest the scaffolds and release DNA from cells, a lysis solution in 1X tris(hydroxymethyl)aminomethane-ethylenediaminetetraacetic acid (Tris-EDTA) buffer (10 mM Tris-HCl, 1 mM EDTA, pH = 7.5, 0.2% Triton X-100) and lysozyme (all Sigma-Aldrich) was added to each sample then incubated at 37°C overnight. Cells were then lysed by freeze-thaw cycles for PicoGreen assay preparation. Cell quantification was determined with the Quant-iT PicoGreen dsDNA Kit according to the manufacturer’s protocol (Life Technologies). A standard curve was generated by serial diluting the supplied λ-DNA standard in TE buffer (1 µg/mL, 100 ng/mL, 10 ng/mL, 1 ng/mL and blank). Sample and standard fluorescence values were determined using a plate reader (Infinite M200, TECAN, Grödig, Austria; 480 nm excitation and 520 nm emission). DNA concentration was determined directly from the fluorescence values and converted to total cell number using the experimentally determined conversion of 2.2 pg DNA/cell.

### Statistics

All statistical analyses were performed using SAS 9.1 (SAS Institute, Cary NC, USA). ANOVA with Tukey’s *post hoc* analysis was performed to detect significant differences between groups. An α level of 0.05 was used to determine significance between groups. Data are reported as mean ± standard deviation (SD).

## Results

### Porous MAC Hydrogel Properties

**Figure 3:**
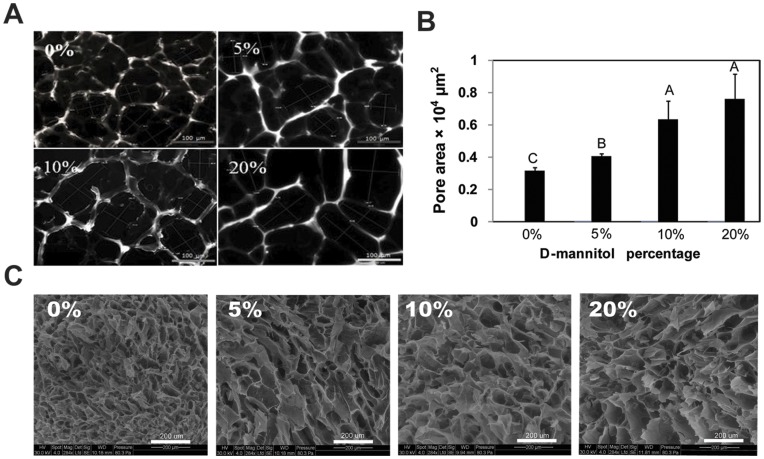
Pore size analysis of MAC scaffolds. (A) Microscope images of acellular MAC scaffolds with varying mass percentages of D-mannitol. (B) Pore sizes of MAC scaffolds with varied D-mannitol percentages. Letters denote significance by single factor ANOVA with Tukey’s *post hoc* analysis (p<0.001). (C) SEM images of MAC scaffolds with varying mass percentages of D-mannitol. Freeze-dried scaffolds collapse during the process, so the pore sizes are not directly comparable to those shown in A and B. Mean ± SD with n = 3.

D-mannitol crystals are easily added to MAC by mixing and crosslinked to form hydrogels. The D-mannitol crystals initially did not noticeably begin to dissolve and turned the gels opaque in appearance (Fig. 1B). D-mannitol quickly dissolved after crosslinked scaffolds were placed in media as observed by the loss of scaffold opacity over time. Crystals fully dissolved one hr after incubation at 37°C. Figure 3A shows microscopy images of pore structures within the hydrogel scaffolds that results from the four different D-mannitol admixing ratios (0, 5, 10, 20 wt%). It is important to point out that cohesive hydrogel scaffolds were difficult to form when mixing more than 20 wt% mannitol by total weight. Hydrogel pore sizes increased with the addition of higher percentages of D-mannitol (Fig. 3B). 0 wt% D-mannitol control scaffolds exhibited an average pore size that was significantly smaller (3150±220 µm^2^) than the other three groups (p<0.001). Average pore sizes significantly increased with D-mannitol admixing percentage and were 4060±160 µm^2^ (p<0.001) for 5 wt%, 6330±1160 µm^2^ (different from 0 and 5%, p<0.001) for 10 wt% and 7600±1550 µm^2^ for 20 wt% D-mannitol scaffolds (different from 0 and 5%, p<0.001). Scaffold samples were freeze dried for two days and SEM images were obtained for each group (Fig. 3C) and confirmed that D-mannitol quickly creates larger pores within MAC hydrogel scaffolds that penetrate into the scaffolds as compared to controls with no D-mannitol added.

### Oxygen Diffusion through Porous Scaffolds Results

**Figure 4:**
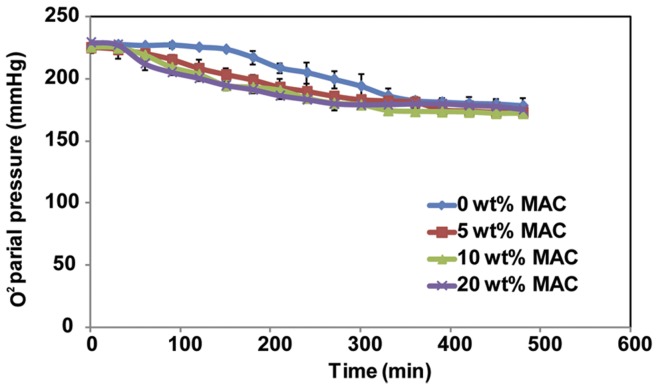
Oxygen diffusion data through 0, 5, 10, 20 wt% MAC scaffolds over 8 hrs. Mean ± SD with n = 3.

Simple unidirectional oxygen diffusion measurements recorded over 8 hrs revealed that scaffolds of increasing porosity (0, 5, 10, 20 wt% D-mannitol) all achieved the same equilibrium oxygen partial pressures (Fig. 4) demonstrating, as expected, that the same amount of oxygen diffused from the top to the bottom chamber through each hydrogel group. What differed was the rate of diffusion in each hydrogel group, with the more porous hydrogels allowing oxygen diffusion to occur faster. Oxygen depletion in the top chamber did not begin until 2.5 hrs in 0 wt% MAC hydrogels, however, more porous 5, 10, and 20 wt% D-mannitol scaffolds started to diffuse oxygen into the bottom chamber within approximately half an hour. A trend was seen toward faster diffusion in more porous scaffolds (more D-mannitol admixed, Fig. 4), but we were limited by our set-up and choice to simulate the static culture environment. Stirring of one or both chambers would have provided a stronger driving force for oxygen diffusion in all groups while potentially providing more separation in the oxygen transport responses of each hydrogel type.

### MAC Scaffold Stiffness and Swelling Results

Since our goal was to use these scaffolds to specify NSPC differentiation, we had to be cognizant of the elastic stiffness of the gels since this is known to significantly affect differentiation [38], [39]. We have shown previously that optimal differentiation into neurons and mature glia is observed when substrate stiffness (E) is on the order of native brain tissue or 0.5–1 kPa [38], [51], [52]. Results from rheology and swelling are presented in Table 1. Elastic stiffness for each group were all in the optimal range for NSPC differentiation, between 0.5 and 0.7 kPa, and were not significantly different from one another by ANOVA (p>0.05). Swelling ratio significantly increased with D-mannitol admixing percentage (MAC compared to MAC with 10 or 20% D-mannitol; p<0.05), indicating that scaffolds with higher percentages of D-mannitol at crosslinking resulted is not only more porous scaffolds but hydrogels with significantly less crosslinks [53].

**Table 1:** Rheology and swelling results

D-Mannitol wt% in MAC	E (Pa) Elastic Modulus	G* (Pa) Complex modulus	Swelling Ratio (Q_M_)
0%	567.99±40.36	189±40.36	9.38±0.31 (B)
5%	628.27±52.52	209±52.52	12.34±0.92 (AB)
10%	659.36±67.98	219±78.6	12.98±1.30 (A)
20%	562.81±108.44	187.6±108.44	13.79±0.54 (A)

Letters denote significance by single factor ANOVA with Tukey’s *post hoc* analysis (p<0.01).

### Immunohistochemistry Results

**Figure 5:**
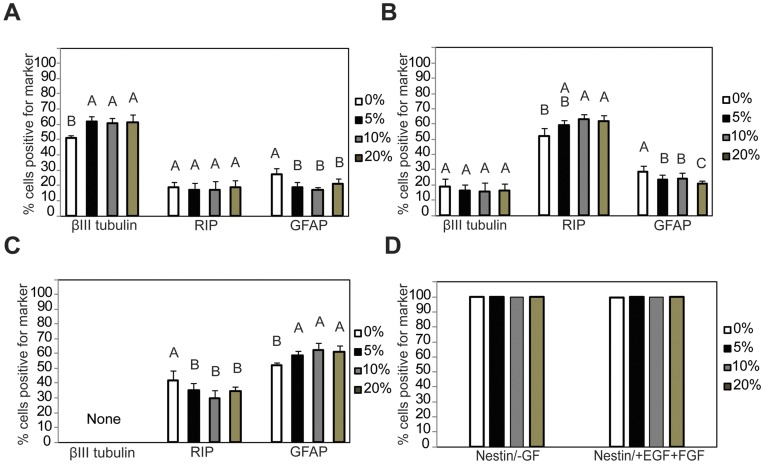
Fluorescence staining results for NSPC differentiation. Quantification of IHC at day 14 shows that more porous scaffolds (up to 20 wt% D-mannitol initially) in (A) neuron specific media (IFN-γ) favor neurons. (B) Oligodendrocyte specific media (PDGF-AA) favor oligodendrocytes and (C) in astrocyte specific media (BMP-2) favor astrocytes. (D) Control media with no growth factors (-GF) as well as with proliferation growth factors (+EGF+FGF) maintain nestin expression (note: error bars are included but too small to see). Letters denote significance by single factor ANOVA (p<0.001). Mean ± SD with n = 3.

**Figure 6:**
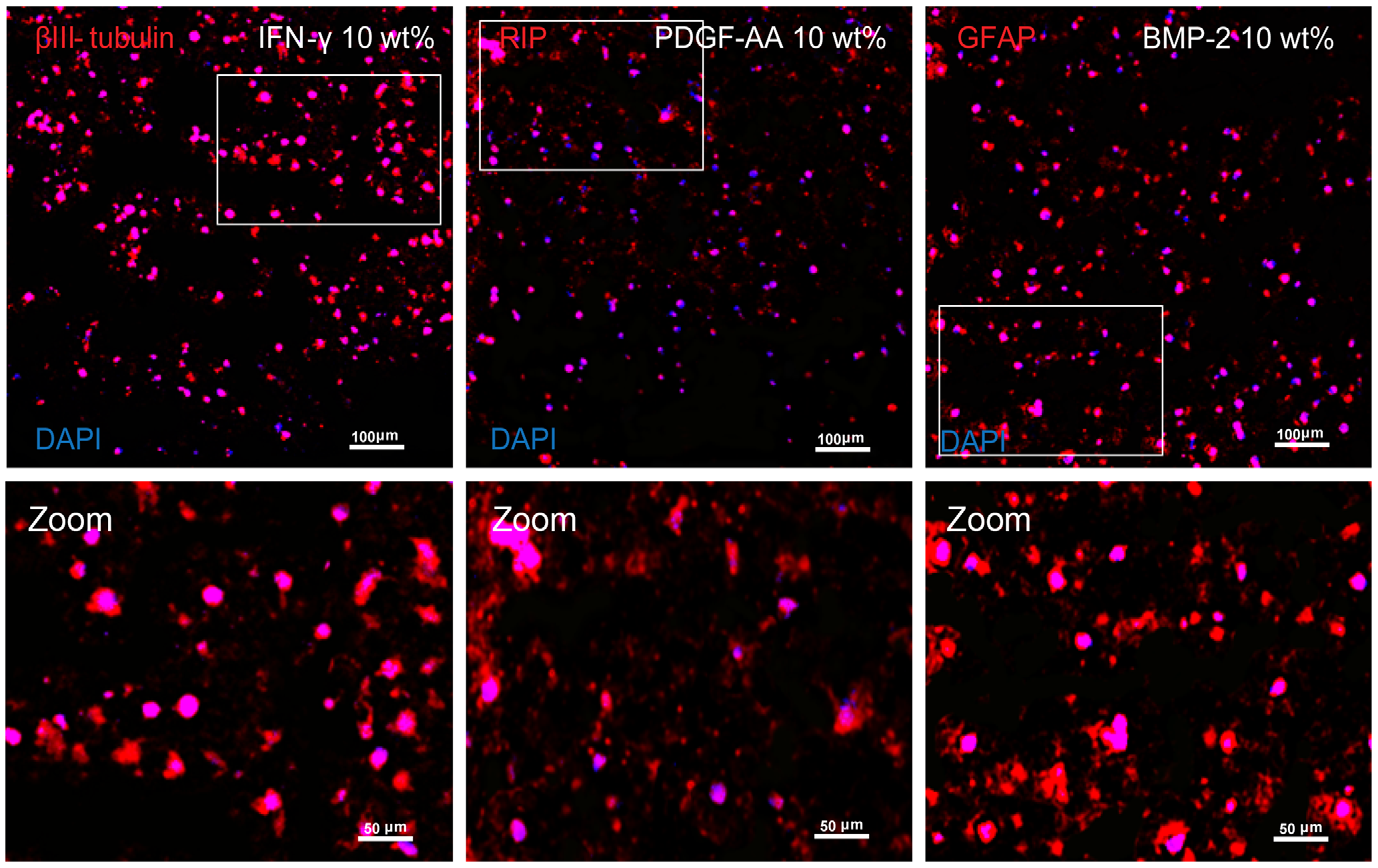
Multiphoton confocal images of fluorescence staining for neurons, oligodendrocytes and astrocytes in 10 wt% scaffolds obtained at the center region of whole scaffolds. Corresponding zoomed regions (white rectangle) for each image are provided below. Nuclei appear blue by Hoechst 33342, cell staining for each differentiation marker appear red by Alexa-Fluor 546.

NSPC differentiation was observed and assayed by IHC in 3D MAC scaffolds (0, 5, 10, 20 wt% D-mannitol) with three different growth factors (150 ng/mL IFN-γ, 25 ng/mL PDGF-AA, 20 ng/mL BMP-2) after 14 d (Figs. 5 and 6). Neuronal differentiation by INF-γ treatment (Fig. 5A) showed that the control group had the lowest percentage of neurons (β-III tubulin positive) among the four groups, and D-mannitol porous gels were all similar (61.7±3.6%, 60.7±3.6%, 61.4±5.0% for 5, 10 and 15 wt% D-mannitol respectively; p>0.05) but significantly higher than the control (50.8±2.2%; p<0.001). Interestingly, control hydrogels contained a significantly higher percentage of GFAP positive cells (27±4%) than the D-mannitol groups (p<0.001). There was no significant difference between the four porosities for the percentage of RIP positive cells with IFN-γ treatment (p>0.05). Oligodendrocyte differentiation stimulation by PDGF-AA (Fig. 5B) showed that 5, 10 and 20 wt% D-mannitol scaffolds resulted in increasing percentages of RIP positive cells with 59.3±3.5%, 63.1±3.2% and 61.8±4.1% respectively; while the control group resulted in 51.8±5.3% RIP positive cells, which was significantly smaller than all D-mannitol containing groups (p<0.001). When treated with PDGF-AA the total percentage of neurons showed no significant difference between the four groups (p>0.05), while the percentage of GFAP positive cells was highest in the control group (28±4%) and decreased as porosity increased (p<0.001). Astrocyte differentiation stimulation by BMP-2 results (Fig. 5C) showed that control group had the lowest GFAP positive cell percentage (51.8±1.8%) compared with the other three groups (58.9±2.7%, 62.5±4.5%, 61.2±4.1% for 5, 10 and 15 wt% D-mannitol respectively; p<0.001) but had a significantly larger RIP positive percentage than scaffolds with D-mannitol (p<0.001). There were no β-III positive cells in any BMP-2 treated hydrogels. No nestin positive staining was observed for all growth factor treatments and porosities, suggesting complete differentiation of NSPCs by 14 d of culture. Representative multiphoton confocal images (Fig. 6) taken at the center of 10 wt % D-mannitol scaffolds show that the majority of cells stain positive for the desired phenotypic markers.

### Total Cell Numbers

**Figure 7:**
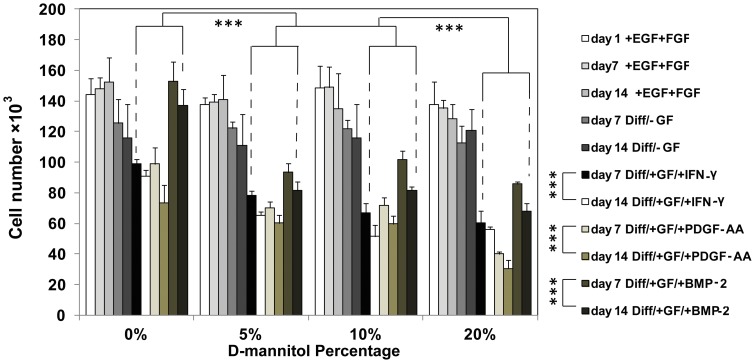
Total cell number at day 7 and 14 for porous scaffolds cultured in control (+EGF+FGF, -GF) and differentiation (INF-γ, PDGF-AA, BMP-2) media. NSPCs were initially seeded at 200 × 10^3^ cells/scaffold. *** denotes significance by two-factor ANOVA (p<0.0001). Mean ± SD with n = 3. All scaffolds were cultured for 1 d in expansion media (+EGF+FGF) then switched to conditions labeled in the caption.

The results of total cell quantification following differentiation to neurons, oligodendrocytes and astrocytes in increasingly porous scaffolds are shown in Figure 7. Interestingly, total cell numbers significantly decreased with increasing porosity (p<0.0001). Total cell number also significantly decreased at day 14 compared to day 7 for all groups (p<0.0001).

## Discussion

MAC hydrogel scaffolds are derived from chitosan, which is distributed widely in insects, invertebrates, fungi and yeasts. Chitosan is FDA approved for wound healing, biocompatible and antibacterial [54]. We have shown previously that MAC scaffolds can serve as a highly tunable growth matrix for NSPCs and can offer an appropriate environment for cell migration, proliferation and differentiation [2], [38].

In this study we present a new technique to enhance the porosity of photocrosslinkable MAC hydrogel scaffolds using a biocompatible porogen D-mannitol during live cell encapsulation. This approach could also be more widely used in photocrosslinked hydrogel systems based on polyethylene glycol, polyethylene oxide or polyacrylamide. Our pore enhancing technique allows us to mix all constituents together (biomaterial, cells, porogen, etc.), mold and crosslink into place with UV light (Fig. 1A). D-mannitol is biocompatible, such that when it is admixed with cells it does not affect normal cell responses. It is important to point out that mannitol is mildly acidic in aqueous conditions and is used to gently increase osmolarity clinically [55]. As we demonstrate, D-mannitol should be fully dissolved and removed from tissue engineered scaffolds before utilization *in vivo* to avoid these potential interactions. One of the most widely accepted techniques to create macropores in scaffolds is solvent casting or salt leaching [21]. Despite its popularity, this technique typically requires sodium chloride as the porogen, thus it cannot be used in the presence of live cells. Dissolution of sodium chloride severely disrupts the osmolality of the culture medium leading to cell rupture and death. Additionally, the dissolution process typically takes at least 24 hrs, thus increasing exposure and likelihood of cell death. A common technique for improving porosity of chitosan based scaffolds is to use snap freezing, however, this is not cell-compatible and does not allow incorporation of cells during construct formation [56], [57]. The main goal of this study was to investigate whether MAC scaffolds with larger average pore sizes enhanced the microenvironment for NSPC support and differentiation. Toward this goal, we selected D-mannitol as a biocompatible porogen and showed it could produce large pores (80–120 µm) within hydrogel scaffolds, in a nontoxic process that lasted less than 1 hr. The addition of higher weight percentages of D-mannitol resulted in enhanced porosity (Fig. 3). We believe that the pore enhancement mechanism results from D-mannitol both interrupting the crosslinking process and inhabiting interior spaces in MAC where finer pore structures would normally form (Fig. 3). The cryosection and SEM images further confirm that when D-mannitol dissolves away, it leaves larger and more interconnected pores inside of the hydrogel. The results from our swelling experiments (Table 1) confirm that the addition of high percentages of D-mannitol results in hydrogels that can swell more because of lower crosslinking densities [53], however, mechanical properties did not decrease due to the inclusion of D-mannitol. Differences could be masked by measurement errors.

Previous studies have reported that cell metabolic state correlates with average pore size in scaffolds [18]. Porcine chondrocytes cultured within chitosan scaffolds improved proliferation and metabolic activity with increased interconnected pore size [58], [59], [60]. Porous photocrosslinked PEG hydrogel scaffolds have been created for the growth of primary embryonic mouse brain neurons by the addition of fibrinogen/thrombin before photocrosslinking, thus creating an interpenetrating network [61]. Pores with average diameters of 0.5–2 µm were achieved by collagenase digestion that allowed some process extension. Due to their small size, these micropores did not allow significant cell migration over the base hydrogel material. The inclusion of fibrin/thrombin followed by enzyme treatment for the creation of pores may be suitable for *in vitro* studies; however, the *in vivo* utility is unclear. For application to CNS tissue engineering, a macroporous scaffold (pores >40 µm) is required and pore formation must be able to proceed quickly and safely after cell inclusion and scaffold formation, which can be achieved with our D-mannitol-MAC approach.

In this study we demonstrate that increased porosity leads to enhanced oxygen diffusion through our constructs. All scaffolds made with D-mannitol began to noticeably diffuse oxygen 2 hrs before MAC control scaffolds in a static environment (Fig. 4). This demonstrates that D-mannitol admixing and dissolution creates scaffolds that allow for faster and more efficient oxygen diffusion generating a favorable environment for cell growth and differentiation. The ability to provide enhanced diffusion should also be important for supporting NSPC activity *in vivo*. Increased porosity increases oxygen diffusion as well as waste and nutrient mass transport. Thus, scaffolds integrating larger pores better maintain an appropriate microenvironment for cells in scaffolds. Tissue engineering requires the formation of large uniformly cell-seeded scaffolds; however, current approaches have difficulty achieving both. The most challenging aspect is supplying all cells with sufficient levels of oxygen and nutrients to support their survival and growth, this is especially true in the center of large scaffolds [9], [62]. As such, hypoxia has been considered as a key limiting factor to scale up 3D *in vitro* scaffolds [63], and more interior scaffold regions have been observed to support lower cell densities, which directly correlates to lower oxygen tensions [8]. Other work has revealed that factors such as nutrient transport and waste removal are equally important for cell survival and differentiation [64]; however, in this study we focused on studying potential oxygen availability.

Results from NSPC differentiation experiments (Figs. 6–7) clearly demonstrate the influence of enhanced porosity during stem cell differentiation. Larger pores in MAC scaffolds promote NSPCs differentiation into neurons, oligodendrocytes, and astrocytes compared to control scaffolds after 14 d (Fig. 6). However, better differentiation in more porous scaffolds corresponded to lower total cell numbers (Fig. 7). These results taken altogether with the results from oxygen diffusion (Fig. 4), suggest to us that the 3D culture environment combined with larger pore structures effectively limits NSPCs proliferation while encouraging cells to differentiate into neurons, astrocytes or oligodendrocytes, depending on the soluble growth factor environment. Most somatic differentiated cells display very limited proliferation potential, and the cell phenotypes we are interested in, such as neurons, loose the capacity for cell division and differentiation once terminal differentiation occurs [65], [66]. Interestingly, increasing the percentage of admixed D-mannitol did not significantly enhance NSPC differentiation, and similar percentages of desired lineages were seen in scaffolds treated with differentiation factors. This is similar to the results seen from our oxygen diffusion measurements (Fig. 2) suggesting that small molecule nutrient/waste diffusion might already be maximized in the range of 5–10 wt% D-mannitol. Additional increases in D-mannitol, and thus pore sizes, does not appreciably enhance diffusion. In our experiments we observed significantly decreasing cellularity only when NSPCs were given differentiation factors (Fig. 5). NSPCs cultured in growth media (EGF and FGF) or control media (no growth factors) maintained cellularity (Fig. 7) and expression of nestin (Fig. 6D). Most likely the observed differences in total cell number when exposed to different differentiation factors are directly related to the activated signaling pathways. Interestingly, PDGF-AA and IFN-γinduced differentiation have both been shown to involve ERK1/2 activation [50], [67]; however, BMP-2 activation involves Smad activation [68]. Previous work has shown that oxygen tension can control proliferation of NSPCs, with high cell numbers seen at lower oxygen tensions [69]. Increasing available oxygen during differentiation enhances the final percentages of mature lineages that are obtained in in vitro monolayer culture [70].

Laminin protein, a well-known ECM protein of the nervous system [71] and NSPC niche, was covalently bound to MAC with the addition of EDC and sulfo-NHS to encourage NSPC survival and differentiation. Laminins can self-assemble, bind to other molecules and laminin-cell interactions are mediated by integrins, dystroglycan and other receptors [72]. Laminin protein has been proven to enhanced NSPC migration, expansion and differentiation [73]. To properly drive differentiation, it is vital to activate integrins, and the crosstalk between integrins and growth factor receptors is required for both stem cell proliferation and differentiation [74]. We believe that our niche inspired approach further allowed us to specifically target desired differentiation responses while limiting proliferation.

It is important to note that none of the MAC formulations deteriorated over the 14 d culture period. MAC is degraded enzymatically by lysozyme through the hydrolysis of glycosidic bonds. MAC hydrogels have been previously shown to degrade to 50% of their initial mass after one month in typical culture conditions [42]. NSPCs effectively remained encapsulated within the MAC portions of the hydrogel and the confocal images reveal that they are just beginning to send out projections at day 14 (Fig. 5), which was difficult to observe due to the autofluorescence of MAC. It is also important to note that the cell encapsulation (Fig. 7) was not sufficient to entrap dsDNA from apoptotic cells that occurred alongside NSPC differentiation, since total DNA quantitation demonstrated reduced cellularity in differentiation factor treated scaffolds. It is possible that network formation and cell-cell interactions are not maximized in these MAC scaffolds. In future studies it would be interesting to culture these scaffolds for longer periods of time, 3–4 wks, to determine if as the MAC matrix disappears the cells remodel the environment effectively migrating and sending out significant process extensions. We could potentially accelerate degradation by adding lysozyme to the culture, or design a new crosslinking system that is degradable by proteolytic enzymes the NSPCs and their progeny secrete. Proteolytic enzymes are integral to the processes of tissue remodeling and formation where migrating cells require active control of the ECM and in the native NSC environment, Matrix metalloproteinases (MMPs) facilitate migration and differentiation [75]. The MMP-1, or collagenase, cleavable sequences have been incorporated into the backbone of photopolymerizable polyethylene glycol allowing MMP-1 mediated cell migration through the hydrogel scaffold [76], [77]. Differentiating NSPCs would most likely require gelatinase cleavable sequences, as MMP-2 and 9 have been shown to be important in neural development and differentiation [75]. MMP-2 has been shown to be particularly important in postnatal development and in migration [78] and axon outgrowth [79].

### Conclusions

NSPCs and other adult stem cells reside in unique niches that provide the cues necessary to direct proliferation and differentiation. This work shows that D-mannitol can be used to enhance the porosity of photopolymerizable MAC hydrogels to improve oxygen diffusion while stimulating NSPC differentiation. In phenotype-specific media these scaffolds efficiently promote NSPCs differentiation into neurons, oligodendrocytes, and astrocytes. Our approach enables creation of porous 3D hydrogel constructs for CNS regeneration and also promotes further understanding of the environmental cues that influence stem cell and differentiation. This work provides a novel, simple and inexpensive approach to create macroporous cell encapsulated scaffolds utilizing a quickly dissolving nontoxic porogen. This affords the potential to supply space for interior cell growth as well space for exogenous cells to penetrate into the scaffold, as is important for future neural tissue engineering applications.

## Supporting Information

Table S1 **NSPC differentiation experiment on laminin coated glass coverslips with soluble BMP-2.** NSPCs were dissociated and cultured for one day in growth medium then 7 d in differentiation medium containing BMP-2 (0, 5 ng/mL, 10 ng/mL, 20 ng/mL, 40 ng/mL or 80 ng/mL). Samples were fixed then stained for glial fibrillary acidic protein (GFAP), imaged and quantified for the percentage of cells staining positive for GFAP as compared to Hoechst 33342 staining.
(DOCX)

## References

[1] Bryant SJ, Cuy JL, Hauch KD, Ratner BD (2007) Photo-patterning of porous hydrogels for tissue engineering. Biomaterials 28: 2978–2986. 17397918 10.1016/j.biomaterials.2006.11.033 PMC1950475

[2] Leipzig ND, Wylie RG, Kim H, Shoichet MS (2011) Differentiation of neural stem cells in three-dimensional growth factor-immobilized chitosan hydrogel scaffolds. Biomaterials 32: 57–64. 20934216 10.1016/j.biomaterials.2010.09.031

[3] Ma Z, Kotaki M, Inai R, Ramakrishna S (2005) Potential of nanofiber matrix as tissue-engineering scaffolds. Tissue Eng 11: 101–109. 15738665 10.1089/ten.2005.11.101

[4] Kim BS, Mooney DJ (1998) Development of biocompatible synthetic extracellular matrices for tissue engineering. Trends Biotechnol 16: 224–230. 9621462 10.1016/s0167-7799(98)01191-3

[5] Sachlos E, Czernuszka JT (2003) Making tissue engineering scaffolds work. Review: the application of solid freeform fabrication technology to the production of tissue engineering scaffolds. Eur Cell Mater 5: 29–39; discussion 39–40. 10.22203/ecm.v005a03 14562270

[6] Ahn G, Park JH, Kang T, Lee JW, Kang HW, et al (2010) Effect of pore architecture on oxygen diffusion in 3D scaffolds for tissue engineering. J Biomech Eng 132: 104506. 20887024 10.1115/1.4002429

[7] Karande TS, Ong JL, Agrawal CM (2004) Diffusion in musculoskeletal tissue engineering scaffolds: design issues related to porosity, permeability, architecture, and nutrient mixing. Ann Biomed Eng 32: 1728–1743. 15675684 10.1007/s10439-004-7825-2

[8] Malda J, Rouwkema J, Martens DE, Le Comte EP, Kooy FK, et al (2004) Oxygen gradients in tissue-engineered PEGT/PBT cartilaginous constructs: measurement and modeling. Biotechnol Bioeng 86: 9–18. 15007836 10.1002/bit.20038

[9] Malda J, Klein TJ, Upton Z (2007) The roles of hypoxia in the in vitro engineering of tissues. Tissue Eng 13: 2153–2162. 17516855 10.1089/ten.2006.0417

[10] O'Brien FJ, Harley BA, Yannas IV, Gibson LJ (2005) The effect of pore size on cell adhesion in collagen-GAG scaffolds. Biomaterials 26: 433–441. 15275817 10.1016/j.biomaterials.2004.02.052

[11] Ji C, Khademhosseini A, Dehghani F (2011) Enhancing cell penetration and proliferation in chitosan hydrogels for tissue engineering applications. Biomaterials 32: 9719–9729. 21925727 10.1016/j.biomaterials.2011.09.003

[12] LiVecchi AB, Tombes RM, LaBerge M (1994) In vitro chondrocyte collagen deposition within porous HDPE: substrate microstructure and wettability effects. J Biomed Mater Res 28: 839–850. 7983082 10.1002/jbm.820280802

[13] Kuberka M, von Heimburg D, Schoof H, Heschel I, Rau G (2002) Magnification of the pore size in biodegradable collagen sponges. Int J Artif Organs 25: 67–73. 11853074 10.1177/039139880202500111

[14] Nehrer S, Breinan HA, Ramappa A, Young G, Shortkroff S, et al (1997) Matrix collagen type and pore size influence behaviour of seeded canine chondrocytes. Biomaterials 18: 769–776. 9177854 10.1016/s0142-9612(97)00001-x

[15] Hing KA, Best SM, Tanner KE, Bonfield W, Revell PA (1999) Quantification of bone ingrowth within bone-derived porous hydroxyapatite implants of varying density. J Mater Sci Mater Med 10: 663–670. 15347983 10.1023/a:1008900127475

[16] Lu JX, Flautre B, Anselme K, Hardouin P, Gallur A, et al (1999) Role of interconnections in porous bioceramics on bone recolonization in vitro and in vivo. J Mater Sci Mater Med 10: 111–120. 15347932 10.1023/a:1008973120918

[17] Hulbert SF MS, Klawitter JJ (1971) Compatibility of porous ceramics with soft tissue: application to tracheal protheses. Journal of Biomechanical Materials Research part A, 5.

[18] O'Brien FJ, Harley BA, Waller MA, Yannas IV, Gibson LJ, et al (2007) The effect of pore size on permeability and cell attachment in collagen scaffolds for tissue engineering. Technol Health Care 15: 3–17. 17264409

[19] Chen GP, Ushida T, Tateishi T (2002) Scaffold design for tissue engineering. Macromolecular Bioscience 2: 67–77.

[20] Nam YS, Yoon JJ, Park TG (2000) A novel fabrication method of macroporous biodegradable polymer scaffolds using gas foaming salt as a porogen additive. J Biomed Mater Res 53: 1–7. 10634946 10.1002/(sici)1097-4636(2000)53:1<1::aid-jbm1>3.0.co;2-r

[21] Liao CJ, Chen CF, Chen JH, Chiang SF, Lin YJ, et al (2002) Fabrication of porous biodegradable polymer scaffolds using a solvent merging/particulate leaching method. J Biomed Mater Res 59: 676–681. 11774329 10.1002/jbm.10030

[22] Zhang R, Ma PX (1999) Poly(alpha-hydroxyl acids)/hydroxyapatite porous composites for bone-tissue engineering. I. Preparation and morphology. J Biomed Mater Res 44: 446–455. 10397949 10.1002/(sici)1097-4636(19990315)44:4<446::aid-jbm11>3.0.co;2-f

[23] Boland ED, Wnek GE, Simpson DG, Pawlowski KJ, Bowlin GL (2001) Tailoring tissue engineering scaffolds using electrostatic processing techniques: A study of poly(glycolic acid) electrospinning. Journal of Macromolecular Science-Pure and Applied Chemistry 38: 1231–1243.

[24] Fabbri M, Celotti GC, Ravaglioli A (1994) Granulates Based on Calcium-Phosphate with Controlled Morphology and Porosity for Medical Applications - Physicochemical Parameters and Production Technique. Biomaterials 15: 474–477. 8080940 10.1016/0142-9612(94)90228-3

[25] Zhang HL, Li JF, Zhang BP (2007) Microstructure and electrical properties of porous PZT ceramics derived from different pore-forming agents. Acta Materialia 55: 171–181.

[26] Tadic D, Beckmann F, Schwarz K, Epple M (2004) A novel method to produce hydroxyapatite objects with interconnecting porosity that avoids sintering. Biomaterials 25: 3335–3340. 14980428 10.1016/j.biomaterials.2003.10.007

[27] Kim J, Yaszemski MJ, Lu LC (2009) Three-Dimensional Porous Biodegradable Polymeric Scaffolds Fabricated with Biodegradable Hydrogel Porogens. Tissue Engineering Part C-Methods 15: 583–594. 19216632 10.1089/ten.tec.2008.0642 PMC2819712

[28] Xu HH, Takagi S, Quinn JB, Chow LC (2004) Fast-setting calcium phosphate scaffolds with tailored macropore formation rates for bone regeneration. J Biomed Mater Res A 68: 725–734. 14986327 10.1002/jbm.a.20093

[29] Takagi S, Chow LC (2001) Formation of macropores in calcium phosphate cement implants. J Mater Sci Mater Med 12: 135–139. 15348319 10.1023/a:1008917910468

[30] Markovic M, Takagi S, Chow LC (2000) Formation of macropores in calcium phosphate cements through the use of mannitol crystals. Bioceramics 192–1: 773–776.

[31] Xu HH, Quinn JB, Takagi S, Chow LC, Eichmiller FC (2001) Strong and macroporous calcium phosphate cement: Effects of porosity and fiber reinforcement on mechanical properties. J Biomed Mater Res 57: 457–466. 11523041 10.1002/1097-4636(20011205)57:3<457::aid-jbm1189>3.0.co;2-x

[32] Xu HH, Weir MD, Burguera EF, Fraser AM (2006) Injectable and macroporous calcium phosphate cement scaffold. Biomaterials 27: 4279–4287. 16650891 10.1016/j.biomaterials.2006.03.001

[33] Straley KS, Foo CW, Heilshorn SC (2010) Biomaterial design strategies for the treatment of spinal cord injuries. J Neurotrauma 27: 1–19. 19698073 10.1089/neu.2009.0948 PMC2924783

[34] Temple S (2001) The development of neural stem cells. Nature 414: 112–117. 11689956 10.1038/35102174

[35] Reynolds BA, Weiss S (1992) Generation of neurons and astrocytes from isolated cells of the adult mammalian central nervous system. Science 255: 1707–1710. 1553558 10.1126/science.1553558

[36] Gage FH (2000) Mammalian neural stem cells. Science 287: 1433–1438. 10688783 10.1126/science.287.5457.1433

[37] Ayuso-Sacido A, Roy NS, Schwartz TH, Greenfield JP, Boockvar JA (2008) Long-term expansion of adult human brain subventricular zone precursors. Neurosurgery 62: 223–229; discussion 229–231. 10.1227/01.NEU.0000311081.50648.4C 18300911

[38] Leipzig ND, Shoichet MS (2009) The effect of substrate stiffness on adult neural stem cell behavior. Biomaterials 30: 6867–6878. 19775749 10.1016/j.biomaterials.2009.09.002

[39] Saha K, Keung AJ, Irwin EF, Li Y, Little L, et al (2008) Substrate modulus directs neural stem cell behavior. Biophys J 95: 4426–4438. 18658232 10.1529/biophysj.108.132217 PMC2567955

[40] Murphy CM, Haugh MG, O'Brien FJ (2010) The effect of mean pore size on cell attachment, proliferation and migration in collagen-glycosaminoglycan scaffolds for bone tissue engineering. Biomaterials 31: 461–466. 19819008 10.1016/j.biomaterials.2009.09.063

[41] Murphy CM, O'Brien FJ (2010) Understanding the effect of mean pore size on cell activity in collagen-glycosaminoglycan scaffolds. Cell Adh Migr 4: 377–381. 20421733 10.4161/cam.4.3.11747 PMC2958613

[42] Yu LM, Kazazian K, Shoichet MS (2007) Peptide surface modification of methacrylamide chitosan for neural tissue engineering applications. J Biomed Mater Res A 82: 243–255. 17295228 10.1002/jbm.a.31069

[43] Brown DM (2004) Drug delivery systems in cancer therapy. Totowa, N.J.: Humana Press. x, 390 p. p.

[44] Kilinc A, Onal S, Telefoncu A (2002) Stabilization of papain by modification with chitosan. Turkish Journal of Chemistry 26: 311–316.

[45] Scott RA, Elbert DL, Willits RK (2011) Modular poly(ethylene glycol) scaffolds provide the ability to decouple the effects of stiffness and protein concentration on PC12 cells. Acta Biomaterialia 7: 3841–3849. 21787889 10.1016/j.actbio.2011.06.054 PMC3185173

[46] Baier Leach J, Bivens KA, Patrick CW Jr, Schmidt CE (2003) Photocrosslinked hyaluronic acid hydrogels: natural, biodegradable tissue engineering scaffolds. Biotechnol Bioeng 82: 578–589. 12652481 10.1002/bit.10605

[47] Baumann N, Pham-Dinh D (2001) Biology of oligodendrocyte and myelin in the mammalian central nervous system. Physiol Rev 81: 871–927. 11274346 10.1152/physrev.2001.81.2.871

[48] Aizawa Y, Leipzig N, Zahir T, Shoichet M (2008) The effect of immobilized platelet derived growth factor AA on neural stem/progenitor cell differentiation on cell-adhesive hydrogels. Biomaterials 29: 4676–4683. 18801569 10.1016/j.biomaterials.2008.08.018

[49] Leipzig ND, Xu C, Zahir T, Shoichet MS (2010) Functional immobilization of interferon-gamma induces neuronal differentiation of neural stem cells. J Biomed Mater Res A 93: 625–633. 19591237 10.1002/jbm.a.32573

[50] Hu JG, Fu SL, Wang YX, Li Y, Jiang XY, et al (2008) Platelet-derived growth factor-AA mediates oligodendrocyte lineage differentiation through activation of extracellular signal-regulated kinase signaling pathway. Neuroscience 151: 138–147. 18093741 10.1016/j.neuroscience.2007.10.050

[51] Gefen A, Margulies SS (2004) Are in vivo and in situ brain tissues mechanically similar? J Biomech 37: 1339–1352. 15275841 10.1016/j.jbiomech.2003.12.032

[52] Taylor Z, Miller K (2004) Reassessment of brain elasticity for analysis of biomechanisms of hydrocephalus. Journal of Biomechanics 37: 1263–1269. 15212932 10.1016/j.jbiomech.2003.11.027

[53] Lin HQ, Kai T, Freeman BD, Kalakkunnath S, Kalika DS (2005) The effect of cross-linking on gas permeability in cross-linked poly(ethylene glycol diacrylate). Macromolecules 38: 8381–8393.

[54] Fujimoto T, Tsuchiya Y, Terao M, Nakamura K, Yamamoto M (2006) Antibacterial effects of chitosan solution against Legionella pneumophila, Escherichia coli, and Staphylococcus aureus. Int J Food Microbiol 112: 96–101. 17045689 10.1016/j.ijfoodmicro.2006.06.003

[55] Polderman KH, van de Kraats G, Dixon JM, Vandertop WP, Girbes AR (2003) Increases in spinal fluid osmolarity induced by mannitol. Crit Care Med 31: 584–590. 12576970 10.1097/01.CCM.0000050287.68977.84

[56] Ma L, Gao C, Mao Z, Zhou J, Shen J, et al (2003) Collagen/chitosan porous scaffolds with improved biostability for skin tissue engineering. Biomaterials 24: 4833–4841. 14530080 10.1016/s0142-9612(03)00374-0

[57] Madihally SV, Matthew HW (1999) Porous chitosan scaffolds for tissue engineering. Biomaterials 20: 1133–1142. 10382829 10.1016/s0142-9612(99)00011-3

[58] Griffon DJ, Sedighi MR, Schaeffer DV, Eurell JA, Johnson AL (2006) Chitosan scaffolds: interconnective pore size and cartilage engineering. Acta Biomater 2: 313–320. 16701890 10.1016/j.actbio.2005.12.007

[59] Ranucci CS, Kumar A, Batra SP, Moghe PV (2000) Control of hepatocyte function on collagen foams: sizing matrix pores toward selective induction of 2-D and 3-D cellular morphogenesis. Biomaterials 21: 783–793. 10721747 10.1016/s0142-9612(99)00238-0

[60] Lien SM, Ko LY, Huang TJ (2009) Effect of pore size on ECM secretion and cell growth in gelatin scaffold for articular cartilage tissue engineering. Acta Biomater 5: 670–679. 18951858 10.1016/j.actbio.2008.09.020

[61] Namba RM, Cole AA, Bjugstad KB, Mahoney MJ (2009) Development of porous PEG hydrogels that enable efficient, uniform cell-seeding and permit early neural process extension. Acta Biomater 5: 1884–1897. 19250891 10.1016/j.actbio.2009.01.036

[62] Volkmer E, Drosse I, Otto S, Stangelmayer A, Stengele M, et al (2008) Hypoxia in static and dynamic 3D culture systems for tissue engineering of bone. Tissue Eng Part A 14: 1331–1340. 18601588 10.1089/ten.tea.2007.0231

[63] Arkudas A, Beier JP, Heidner K, Tjiawi J, Polykandriotis E, et al (2007) Axial prevascularization of porous matrices using an arteriovenous loop promotes survival and differentiation of transplanted autologous osteoblasts. Tissue Eng 13: 1549–1560. 17518756 10.1089/ten.2006.0387

[64] Den Buijs JO, Dragomir-Daescu D, Ritman EL (2009) Cyclic deformation-induced solute transport in tissue scaffolds with computer designed, interconnected, pore networks: experiments and simulations. Ann Biomed Eng 37: 1601–1612. 19466547 10.1007/s10439-009-9712-3 PMC4208723

[65] Cassimeris L, Lingappa VR, Plopper G, Lewin B (2011) Lewin's cells. Sudbury, Mass.: Jones and Bartlett Publishers. xxiv, 1053 p. p.

[66] Currais A, Hortobagyi T, Soriano S (2009) The neuronal cell cycle as a mechanism of pathogenesis in Alzheimer's disease. Aging-Us 1: 363–371. 10.18632/aging.100045 PMC2806021 20157524

[67] Song JH, Wang CX, Song DK, Wang P, Shuaib A, et al (2005) Interferon gamma induces neurite outgrowth by up-regulation of p35 neuron-specific cyclin-dependent kinase 5 activator via activation of ERK1/2 pathway. J Biol Chem 280: 12896–12901. 15695523 10.1074/jbc.M412139200

[68] Nakashima K, Takizawa T, Ochiai W, Yanagisawa M, Hisatsune T, et al (2001) BMP2-mediated alteration in the developmental pathway of fetal mouse brain cells from neurogenesis to astrocytogenesis. Proc Natl Acad Sci U S A 98: 5868–5873. 11331769 10.1073/pnas.101109698 PMC33305

[69] Pistollato F, Chen HL, Schwartz PH, Basso G, Panchision DM (2007) Oxygen tension controls the expansion of human CNS precursors and the generation of astrocytes and oligodendrocytes. Molecular and Cellular Neuroscience 35: 424–435. 17498968 10.1016/j.mcn.2007.04.003

[70] Santilli G, Lamorte G, Carlessi L, Ferrari D, Rota Nodari L, et al (2010) Mild hypoxia enhances proliferation and multipotency of human neural stem cells. PLoS One 5: e8575. 20052410 10.1371/journal.pone.0008575 PMC2797394

[71] Lia SC (2005) β1 Intergrins and neural stem cells: making sense of extracellular environment. Bioessays 27: 697–707. 10.1002/bies.20256 15954093

[72] Colognato H, Yurchenco PD (2000) Form and function: The laminin family of heterotrimers. Developmental Dynamics 218: 213–234. 10842354 10.1002/(SICI)1097-0177(200006)218:2<213::AID-DVDY1>3.0.CO;2-R

[73] Flanagan LA RL, Derzic S, Schwartz PH, Monuki ES (2006) Regulation of human neural precusor cells by laminin and integrins. J Neurosci Res 83: 845–856. 16477652 10.1002/jnr.20778 PMC2409144

[74] Streuli CH (2009) Integrins and cell-fate determination. J Cell Sci 122: 171–177. 19118209 10.1242/jcs.018945 PMC2714415

[75] Tonti GA MF, Cacci E, Biogioni S (2009) Neural stem cells at the crossroads: MMPs may tell the Int J Dey Biol. 53: 1–17. 10.1387/ijdb.082573gt 19123122

[76] Lee SH, Moon JJ, Miller JS, West JL (2007) Poly(ethylene glycol) hydrogels conjugated with a collagenase-sensitive fluorogenic substrate to visualize collagenase activity during three-dimensional cell migration. Biomaterials 28: 3163–3170. 17395258 10.1016/j.biomaterials.2007.03.004

[77] West JL, Hubbell JA (1999) Polymeric biomaterials with degradation sites for proteases involved in cell migration. Macromolecules 32: 241–244.

[78] Ogier C, Bernard A, Chollet AM, Le Diguardher T, Hanessian S, et al (2006) Matrix metalloproteinase-2 (MMP-2) regulates astrocyte motility in connection with the actin cytoskeleton and integrins. Glia 54: 272–284. 16845676 10.1002/glia.20349

[79] Zuo J, Ferguson TA, Hernandez YJ, Stetler-Stevenson WG, Muir D (1998) Neuronal matrix metalloproteinase-2 degrades and inactivates a neurite-inhibiting chondroitin sulfate proteoglycan. Journal of Neuroscience 18: 5203–5211. 9651203 10.1523/JNEUROSCI.18-14-05203.1998 PMC6793496

