# Supplementary material for: 3D Differentiation of Neural Stem Cells in Macroporous Photopolymerizable Hydrogel Scaffolds
Source: PLoS One. 2012 Nov 7;7(11):e48824. doi: 10.1371/journal.pone.0048824 (PMC3492243; doi:10.1371/journal.pone.0048824)
Supplement: Table S1 — NSPC differentiation experiment on laminin coated glass coverslips with soluble BMP-2. NSPCs were dissociated and cultured for one day in growth medium then 7 d in differentiation medium containing BMP-2 (0, 5 ng/mL, 10 ng/mL, 20 ng/mL, 40 ng/mL or 80 ng/mL). Samples were fixed then stained for glial fibrillary acidic protein (GFAP), imaged and quantified for the percentage of cells staining positive for GFAP as compared to Hoechst 33342 staining. (DOCX) [file pone.0048824.s001.docx]

Supplementary Table 1.

| BMP-2 Concentration (ng/mL) | GFAP positive percentage (%) |
| --- | --- |
| 0 | 0.5 ± 1 (C) |
| 5 | 95 ± 1 (B) |
| 10 | 100 ± 0.5 (A) |
| 20 | 100 ± 0.3 (A) |
| 40 | 100 (A) |
| 80 | 100 (A) |

Letters denote significance by single factor ANOVA with Tukey’s *post hoc* analysis (p<0.001)
